# Feedback improves compliance of pressure relief activities in wheelchair users with spinal cord injury

**DOI:** 10.1038/s41393-020-0522-7

**Published:** 2020-07-21

**Authors:** Michèle Hubli, Roland Zemp, Urs Albisser, Franziska Camenzind, Olena Leonova, Armin Curt, William R. Taylor

**Affiliations:** 1 Spinal Cord Injury Center, Balgrist University Hospital, University of Zurich, Zurich, Switzerland; 2 Department of Health Sciences and Technology, Institute for Biomechanics, Swiss Federal Institute of Technology in Zurich (ETHZ), Zurich, Switzerland

**Keywords:** Patient education, Spinal cord diseases, Risk factors

## Abstract

**Study design:**

Prospective cross-sectional pre-post pilot study.

**Objectives:**

This pilot study aimed to evaluate the potential for improving pressure relief behaviour in wheelchair users with spinal cord injury (SCI) using a novel feedback system based on textile pressure sensor technology.

**Setting:**

In- and out-patient clinic of the Spinal Cord Injury Center, Balgrist University Hospital, Zurich, Switzerland.

**Methods:**

Nine wheelchair users with SCI (3 females, 50 ± 12 years of age, 2 tetra- and 7 paraplegics) were equipped with a feedback system (*sensomative*_*wheelchair*_) for three continuous weeks. The system consists of a textile pressure mat and a mobile smartphone application that reminds participants to perform missing pressure reliefs during regular and unobserved wheelchair usage in a customized manner. Pressure reliefs were detected using a subject-specific random forest classifier. Improvements of relief quality, duration and frequency were analysed by comparing week 1 (baseline) with no feedback, i.e., only pressure data recorded, against week 2 (with feedback). Carry-over effects of improved relief behaviour were studied in week 3 (no feedback, pressure data only recorded).

**Results:**

All participants increased their relief frequency and performed in median 82% (IQRs: 55%–99%) of the required reliefs while using the feedback system, whereas the median relief frequency was only 11% (IQRs: 10%–31%) during the baseline condition. Every participant who did not perform reliefs of sufficient duration (based on the recommendations of the therapist) during week 1 showed a significant improvement while using the feedback system.

**Conclusion:**

Subject-specific feedback using the novel feedback system may have the potential for improving the regularity of an individual’s relief activities, and may ultimately be an instrument for reducing the risk of developing pressure ulcers.

## Introduction

Individuals with spinal cord injury (SCI) who are confined to a wheelchair are at perpetually high risk of developing pressure ulcers (PUs), which are defined as “a localized injury to the skin and/or underlying tissue usually over a bony prominence as a result of pressure or pressure in combination with shear and/or friction” [1].

During initial acute hospitalisation, 27–40% of individuals with SCI will experience a PU [2–4]. Even after discharge it is the most common secondary complication reported at annual follow-ups [5], and PUs are reported to account for a disproportionate number of rehospitalisation days [6, 7]. The annual prevalence rate is reported to lie within 10 to 38% [8–10], while it is estimated that 50–80% of individuals with SCI may develop a PU at least once in their lifetime [3, 11].

Apart from PUs severely impairing the physical, psychological and social well-being of affected individuals, they may also negatively impact rehabilitation, prevent work or school attendance, and finally delay the return to independent living [3]. PUs account for roughly one-quarter of the cost of care following SCI [9], with the overall annual costs in Germany estimated to be between 1 and 2 billion Euros per year (http://dekubitus.de/dekubitus-entstehung.htm). As a result, health professionals focus their attention on preventing PUs more than on any other secondary complication following SCI [12] by reducing risk factors for PU development. In general, there is no single risk factor that can explain the development of PUs [13]. However, the greatest risk factors seem to be associated with the individual’s mobility/activity and factors negatively influencing blood perfusion, such as diabetes, vascular disease, reduced blood pressure, smoking, or oedemas [13]. Furthermore, the microclimate of the skin, i.e., temperature, humidity and airflow next to the skin surface, is known to modify indirect risk factors for PUs [14, 15]. Recent scientific literature has stressed the presence of both physiological and behavioural risk factors [16], such as reduced self-managed care, and thus effective PU prevention should embrace these critical elements. With regard to the SCI population, many factors interact to predispose an individual to pressure-related skin breakdown. PU formation following SCI is a complex process that is still not completely understood, and recent reviews have highlighted altered pathophysiological processes precipitating PUs in skin that has been denervated due to SCI [17]. Whilst shear forces and friction cause direct skin injuries, prolonged pressure, for example during sitting in wheelchairs, reduces perfusion, and hence deteriorates tissue viability. The combination of prolonged pressure together with shear forces, however, is widely considered to be the key underlying trigger that initiates PU development [18]. Therefore, prolonged wheelchair usage in the SCI population accounts for the high prevalence of seating-acquired PUs covering the ischial tuberosities or sacrum.

Consequently, preventive PU measures include sitting properly (symmetrically, no wrinkles in the clothing etc.), avoiding increased pressure in high-risk regions such as the buttocks, performing regular pressure relieving activities, skin inspection, and using specialized equipment (skin protection wheelchair cushion, customised seat shell, etc.) [19].

After being confined to a wheelchair, individuals with SCI, their family, and caregivers are all educated in PU risk factors, pressure relief strategies, and skin inspection/care processes [20]. Instructions on how to correctly perform pressure reliefs or weight shifts are a routine part of the rehabilitation process. Clinical guidelines vary somewhat, but collectively recommend that participants perform pressure reliefs for 15–60 s every 15–60 min [1, 21, 22] in order to improve tissue reperfusion and maintain tissue integrity [23]. Nevertheless, sustaining long-term adherence to recommended measures in order to prevent PUs remains a major concern. For example, van Loo and colleagues [24] reported that less than 50% of wheelchair users with SCI living at home regularly perform pressure relief activities. This compliance to preventive measures also tends to decrease over time in individuals living at home [24].

In order to increase long-term adherence to PU preventive measures and to support wheelchair users in performing pressure relief activities of sufficient quality (duration and intensity of the relief), sensomative GmbH (Rothenburg, Switzerland) has further developed the textile pressure distribution sensor [25] to a sensor-based solution for monitoring sitting behaviour, consisting of a textile pressure mat (placed underneath the skin protection wheelchair cushion), a data acquisition/transmission unit and a mobile smartphone application. The so-called *sensomative*_*wheelchair*_ technology aims to assist wheelchair users with SCI in preventing PUs by detecting prolonged static pressure periods and providing user feedback to promote the regular and appropriate performance of pressure relief activities. To our knowledge, there is currently only one feedback system for wheelchair users available on the market. MisterGaspard (Captiv, Nantes, France) is a sensor mat, to be place underneath the wheelchair cushion, which is connected to a smartphone application using Bluetooth Low Energy (BLE). However, so far no scientific evaluation of this system has been performed.

The objective of this pilot study was to evaluate the potential of the novel feedback system for preventing PUs by altering the quality and frequency of pressure relief activities.

## Methods

### Participants

Participants with SCI were recruited by an occupational therapist (OT) at the Spinal Cord Injury Center, Balgrist University Hospital, Zurich, Switzerland. They were required to be at least 18-year old, and a wheelchair user in an acute, sub-acute or chronic stage of SCI, and, hence, at a perpetual risk of developing a PU due to the inability to stand or walk, together with diminished sensation below the spinal lesion level. The aetiology of the SCI could either be traumatic or non-traumatic. Participants were excluded if they suffered from major untreated mental health issues (e.g. psychosis or depression), or showed major cognitive, communication, and/or comprehension deficits. This study was approved by the local ethics committee, and all participants provided written informed consent prior to the measurements.

### Study design

**Fig. 1:**
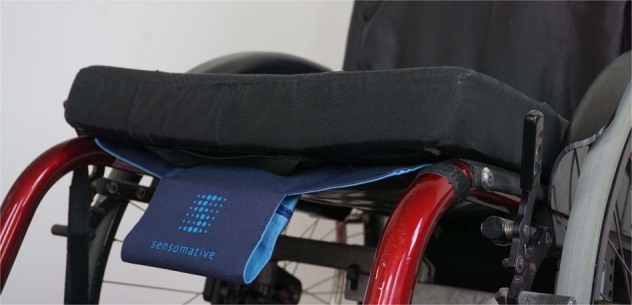
Textile pressure system. Textile pressure mat of the feedback system (placed underneath the skin protection wheelchair cushion) with the data acquisition/transmission unit (located in the small flap with the company logo).

In order to analyse the efficacy of the feedback on the performance of pressure relief activities, sitting behaviour under three different conditions was measured (baseline, feedback, follow-up) with a duration of one week per condition. At the beginning of the first measurement week, the wheelchairs of the participants were equipped with a textile pressure system (see the section “Feedback system”, Fig. 1), which allowed continuous data collection during the entire three weeks measurement period (24 h per day).

At the beginning of the second measurement week, participants were provided with a mobile phone (Nexus 5X, Google, LG, Seoul, Korea) and introduced to the feedback system application by the OT. Firstly, they were guided through the setup process whereby the different possible relief activities, the relief durations, as well as the relief interval, were defined according to the subject’s physical abilities and needs, based on the therapist’s recommendations (Table 1). Subsequently, participants were asked to perform the different relief activities, three times each, under supervision. The different relief positions as well as the upright position were recorded as part of the setup process. After completing the settings process and introducing the different functions of the mobile application to the participants, the feedback system was ready to be used without any further guidance. In contrast to the first and third measurement week, feedback was provided to the participants using the feedback system during the entire second measurement week.

**Table 1:** Characteristics of the nine study participants.

Subject	Sex	Age	Years in wheelchair	Neurological level of injury	AIS	History of PU
1	f	75	14	Th10	A	1
2	m	57	12	Th1	A	1
3	m	58	10	Th6	A	1
4	m	54	1	C4	C	0
5	f	39	9	Th8	A	0
6	m	43	21	Th8	A	0
7	m	40	20	C6	A	1
8	f	35	4	C4	C	1
9	m	51	28	Th6	A	0

History of PU, 1 = yes, 0 = no.

*AIS* ASIA (American Spinal Injury Association) Impairment Scale, *PU* pressure ulcer.

The participants’ sitting behaviour was measured in the third week (follow-up) without any feedback to reveal any learning or washout effects from week two by comparing the outcome with the baseline measurement. At the beginning of the third week, the smartphones with the feedback system were collected and the sitting/relief behaviour of the participants was again recorded only using the textile pressure mat.

### Feedback system

The feedback system (sensomative GmbH, Rothenburg, Switzerland) consists of a textile pressure mat (Fig. 1), a data acquisition/transmission unit and a smartphone application. The textile pressure mat features a thickness of 2 mm, incorporating 8 piezo-resistive textile sensors, each with a size of 4 cm × 4 cm, which are evenly distributed over an area of 35 cm × 35 cm. The data acquisition/transmission unit, located in a small flap hanging to the side of the seat-pan, acquires the pressure data with a frequency of 1 Hz (pressure range: 0–60 kPa, resolution: 8bits) and transmits the data to a connected smartphone using Bluetooth Low Energy (BLE). In addition, the pressure data are stored on a local SD card, in order to analyse each participant’s sitting behaviour, independently of the smartphone application. Central to the system, the smartphone application collects and processes the raw pressure data to provide feedback on pressure relief activities. The participants’ sitting behaviours and the sitting/relief positions are detected continuously using data mining algorithms (see the “Data pre-processing” section). In order to provide highly individual and customised recommendations to users, the required relief duration, the required relief interval, as well as the possible relief techniques (“to the left/right”, “leaning forward”, “lift off”, “seat tilt”) are defined prior to using the feedback system. These goals are set individually and in accordance with each user’s specific needs, pathologies and physical skills, together with the OT. During calibration, the system is additionally customised to account for individual characteristics by assessing the user’s recommended relief techniques (with the OT recommended intensity) and an upright reference sitting position. As a consequence, while using the feedback system, pressure reliefs performed with a lower intensity than the individual recommendation are detected as upright sitting.

After successfully completing the setup process, the feedback system is ready to be used. Users are then provided with information pertaining to their set goals per day, as well as the number of pressure reliefs already performed on the current day. For confirmation of correct functionality, the system additionally displays the current sitting position. If the wheelchair user performs a relief, a pop-up message shows the time remaining to complete each pressure relief activity correctly and of sufficient duration, which is indicated by a sound and a vibration signal. If no successful relief activity is performed, users are notified after a quarter of the predefined relief interval. They are subsequently reminded repeatedly and with an increasing level of disturbance:

- First feedback level: text only notification when no pressure relief is detected for 5/4 of the predefined relief interval (e.g. if the predefined relief interval is 20 min, the first text notification feedback will be sent after 25 min).
- Second feedback level: text notification and an additional vibration signal (after no relief for 6/4 of relief interval).
- Third feedback level: text notification, vibration signal and an additional alarm (after no relief for 7/4 of relief interval); repeated every 1/4 of relief interval until successful relief completion.

### Data pre-processing

Pressure relief activities were detected and feedback was provided in real-time when the feedback system was active (i.e. during the second measurement week). Since all relevant analyses for this study were performed based on the raw pressure data provided by the sensor mat, the relief detection algorithm of the feedback system within the smartphone application was also retrospectively replicated for periods when no direct feedback was provided. Thus, raw pressure data from the local SD card (of the entire measurement period) as well as the recorded upright and relief positions from the mobile phone (at the beginning of the second measurement week) were transferred to a computer where data analysis was performed using MATLAB (vR2018a, MathWorks Inc., Natick, USA). Sitting and relief positions during the three measurement weeks were identified using supervised learning with a random forest classifier [26, 27]. An individual classifier was generated for every subject using the recordings of the different relief techniques (three measurements each) as well as the upright sitting position acquired during the setup process (similarly to [28]). For every subject-specific classifier an ensemble of 200 bagged decision trees was created while using 60% of the input data for growing each tree (MATLAB’s parameter: *InBagFraction* = 0.6). All other parameters were kept at MATLAB’s default levels. To quantify the misclassification probability of the trained classifier, the out-of-bag error was calculated for every subject using the in-built MATLAB function (*oobError)*. In order to classify the sitting position, the subject-specific random forest classifier was applied to the raw pressure data of every single time point during the three-week measurement period. Detected relief positions that lasted longer than 5 s were considered as a relief activity attempt, while reliefs that lasted at least as long as the defined minimum relief duration were ranked as successful reliefs. The relief activity “to the left/right” was only considered if the wheelchair user performed both “to the left” and “to the right” with a time gap of less than 2 min between the two. For calculating the corresponding relief duration, the shorter of the two (“to the left”, “to the right”) was used. All time points with a maximal pressure value higher than 5kPa wereincluded in the calculation of daily sitting time.

The percentage relief duration parameter *Duration*_*Relief*_ was determined by calculating the average relief duration of all relief attempts (including successful reliefs) for every measurement day and dividing it by the defined required relief duration. If participants performed less than three reliefs within one measurement day, the corresponding day was ignored.

The relief frequency parameter *Freq*_*Relief*_ was calculated by dividing the daily number of successful reliefs by the number of required relief activities (calculated using the predefined relief frequency and the measured daily sitting time in the wheelchair).

$$\begin{array}{l}Freq_{Relief} = \frac{{\# Relief_{successful}}}{{\# Relief_{required}}} = \frac{{\# Relief_{successful}}}{{Freq_{ReliefRequired}[\mathrm{min}^{ - 1}] \,\times\, SittingTime[\mathrm{min}]}}\\ \, = \frac{{\# Relief_{successful}}}{{\left( {Invterval_{ReliefRequired}[\mathrm{min}]} \right)^{ - 1} \,\times\, SittingTime[\mathrm{min}]}}\end{array}$$

The median values of the two parameters *Duration*_*Relief*_ and *Freq*_*Relief*_ were calculated for the three measurement weeks for all participants. The median value for the parameter *Duration*_*Relief*_ was not calculated if there were less than three valid measurement days within one of the three measurement weeks (indicated as “NaN” in Fig. 3).

### Statistical analysis

All statistical analyses were performed using IBM SPSS Statistics (v24, SPSS Inc., Chicago, USA) and statistical significance was defined at *p* < 0.05.

Given the limited number of subjects as well as visual inspection of the corresponding Q-Q-Plots for the parameters *Duration*_*Relief*_ and *Freq*_*Relief*_ non-parametric analyses were performed. In order to analyse the influence of the feedback system on relief duration and frequency, a Friedman test was applied. Here, the two parameters *Duration*_*Relief*_ and *Freq*_*Relief*_ were used as the dependent variables and the three conditions (baseline, feedback, follow-up) as the grouping. For the *Duration*_*Relief*_ parameter, only the data of participants with a first week median of less than the predefined relief duration (i.e. those considered to underperform their required relief duration) were considered for the Friedman test, as participants with a sufficient relief duration (even without using a feedback system) were not required to increase the relief duration time. If the Friedman test was significant, a Wilcoxon signed-rank post hoc test was performed, whereby the significance level was Bonferroni-adjusted (*p* < 0.017).

## Results

In total, nine participants with SCI (3 females, 2 tetra- and 7 paraplegics) with a mean age of 50 ± 12 years were recruited (Table 1). One subject (#4) used an electric wheelchair and was only able to perform a passive “seat tilt”. All other subjects were able to actively perform pressure reliefs.

The misclassification probability (out-of-bag error) for the nine subject-specific Random Forest classifiers was between 0% and 7% (average: 2%).

### Relief frequency

**Fig. 2:**
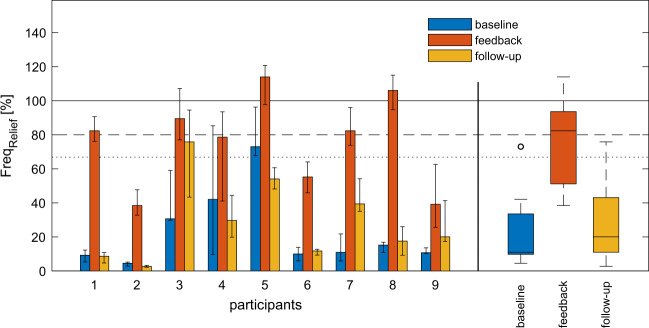
Median relief frequency parameter (***Freq***_***Relief***_) for the three measurement weeks (baseline, feedback, follow-up) calculated based on the performed successful relief activities divided by the predefined (OT recommended) relief number. The error bars indicate the first and third quartiles. The three boxplots on the right summarise all 9 participants for the different measurement weeks. The required relief frequency is highlighted with a solid line (100%), whereas the dashed (80%) and the dotted line (67%) represent the first and second feedback levels.

All participants showed an increased relief frequency (*Freq*_*Relief*_) when using the feedback system compared to both the baseline and follow-up conditions (Fig. 2, Table 2). The Friedman test showed statistical significance in *Freq*_*Relief*_ between measurement weeks (*χ*^2^(2) = 13.556, *p* = 0.001). Post hoc analysis using the Wilcoxon signed-rank test (Table 2) indicated a moderate effect between both the baseline (median (IQR): 11% (10–31%)) and the feedback (82% (55–90%); *r* = −0.628), as well as between the feedback and the follow-up (20% (12–39%); *r* = −0.628) conditions, but not between the baseline and the follow-up (*r* = −0.154).

**Table 2:** Post hoc Wilcoxon signed-rank tests for the relief frequency parameter (*Freq*_*Relief*_) and the three different measurement conditions (baseline, feedback, follow-up) with the corresponding required relief interval, median sitting time and relief frequency (*Freq*_*Relief*_) with the equivalent relief interval time for all participants and the measurement conditions.

Participants	Required relief interval [min]	Median sitting time [h]	*Freq*_*Relief*_ [%]			Relief interval [min]		
			Baseline	Feedback	Follow-up	Baseline	Feedback	Follow-up
1	40	13.2	9	82	9	431	49	460
2	10	7.0	5	38	3	219	26	368
3	50	6.1	31	90	76	163	56	66
4	60	10.1	42	79	30	143	76	202
5	50	8.7	73	114	54	68	44	92
6	50	14.1	10	55	12	502	91	425
7	35	5.4	11	82	39	319	42	89
8	30	12.5	15	106	18	197	28	171
9	50	7.6	11	39	20	467	128	249
Median (IQR)	50 (35–50)	8.7 (7.0–12.5)	11 (10–31)	82 (55–90)	20 (12–39)	219 (163–431)	49 (42–76)	202 (92−368)

				∆ Baseline–feedback	∆ Baseline–follow-up	∆ Feedback–follow-up		
			Z	−2.666	−0.652	−2.666		
			*p*-value	0.008	0.515	0.008		
			Effect size (r)	−0.628	−0.154	−0.628		

The mean ranks for the three different measurement conditions were 1.44 (baseline), 3.00 (feedback) and 1.56 (follow-up) and the level for significance was set at *p* < 0.017 (Bonferroni-corrected). IQR (interquartile range). Effect size: $r = \frac{Z}{{\sqrt N }}$.

### Relief duration

**Fig. 3:**
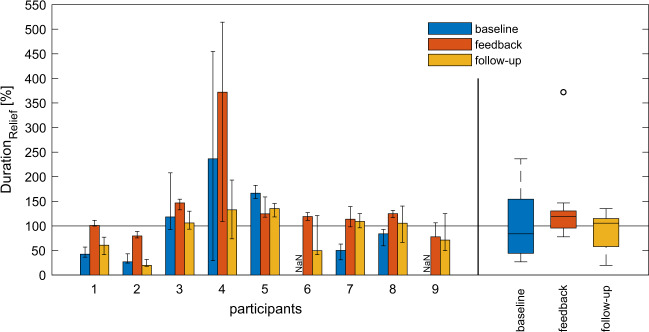
Median relief duration of the three measurement weeks (baseline, feedback, follow-up) in percentage of their OT’s recommended relief duration (***Duration***_***Relief***_). The error bars indicate the first and third quartiles. The three boxplots on the right summarise all nine participants for the different measurement weeks. The OT’s recommended relief duration is highlighted with a solid line (100%).

Two out of nine participants did not perform a sufficient number of reliefs during the first measurement week (baseline), and thus the median value for their *Duration*_*Relief*_ was not calculated. All participants with a relief duration of less than the OT’s recommended relief duration (solid line at 100%) in the first measurement week increased their *Duration*_*Relief*_ while using the feedback system (Table 3/Fig. 3: participants 1/2/7/8). Three participants already showed a sufficient relief duration in week 1 (Fig. 3: participants 3/4/5). Note that subject 4 used an electronic wheelchair and performed only prolonged backward tilting as his relief technique.

**Table 3:** Post hoc Wilcoxon signed-rank tests for the relief duration parameter (*Duration*_*Relief*_) and the three different measurement conditions (baseline, feedback, follow-up) with the corresponding required relief duration, relief duration (*Duration*_*Relief*_) in percentage of the required relief duration as well as with the equivalent relief duration in seconds for all participants and the measurement conditions.

Participants	Required relief duration [s]	*Duration*_*Relief*_ [%]			Relief duration [s]		
		Baseline	Feedback	Follow-up	Baseline	Feedback	Follow-up
1	30	42	101	61	13	30	18
2	60	27	79	20	16	48	12
3	15	118	147	106	18	22	16
4	120	237	372	133	284	446	159
5	30	167	125	135	50	37	41
6	20	NaN	119	50	NaN	24	10
7	30	50	114	109	15	34	33
8	20	84	125	105	17	25	21
9	30	NaN	78	71	NaN	23	21
Median (IQR)	30 (20–30)	84 (46–142)	119 (101–125)	105 (61–109)	17 (16–34)	30 (24–37)	21 (16–33)

		∆ Baseline–feedback	∆ Baseline–follow-up	∆ Feedback–follow-up			
	Z	−1.826	−1.461	−2.201			
	*p*-value	0.068	0.144	0.028			
	Effect size (r)	−0.646	−0.517	−0.635			

Only data of participants with a first week median of less than the predefined relief duration (participant 1, 2, 6–9) were used for the post hoc Wilcoxon signed-rank tests. The mean ranks for the three different measurement conditions were 1.25 (baseline), 3.00 (feedback) and 1.75 (follow-up) and the level for significance was set at *p* < 0.017 (Bonferroni-corrected). IQR (interquartile range). Effect size: $r = \frac{Z}{{\sqrt N }}$.

The Friedman test indicated statistical significance for *Duration*_*Relief*_ between measurement weeks (*χ*^2^(2) = 6.500, *p* = 0.039). The post hoc Wilcoxon signed-rank analysis (Table 3) suggested a moderate effect between any of the three comparisons (−0.646 < *r* < −0.517).

## Discussion

The objective of this pilot study was to evaluate the potential for preventing PUs in wheelchair users with SCI by improving their pressure relief behaviour using a novel feedback system. The results clearly indicate that by using the feedback system, the frequency and duration of pressure relief behaviour was improved for all participants.

Although none of the participants performed the OT’s recommended number of reliefs in week 1, all of them benefited from the feedback system in terms of successful reliefs performed per day, as indicated by a significantly increased relief frequency. The therapist’s recommendation regarding relief frequency and duration was individually customised to the participant’s functional capacity and specific needs. The recommended relief interval in our study was spread between performing a relief every 10 up to 60 min. The median relief frequency for week 1 (baseline, without feedback system) was, however, below 20% of the OT’s recommended relief frequency. This is in line with previous reports suggesting that even when wheelchair users with SCI train for correct relief behaviour with clinicians or therapists, they often only perform a fractional amount of the recommended reliefs during daily life, and these are often with shorter duration and of lower quality [29, 30]. Furthermore, it has recently been shown that wheelchair users do not develop a sufficiently functional pressure relief routine, either in frequency or duration, including weight shifts or other functional in-seat movements [31]. This illustrates the complexity of assigning causation of PU occurrence to the seated behaviour of wheelchair users, and identifies the need for improved clinical techniques designed to develop a functional routine behaviour towards preventing PUs. Here, we show for the first time that by providing suitable feedback to wheelchair users with SCI, such relief behaviour can be positively influenced. In our study, this improvement was indicated by an on average fourfold increase in relief frequency compared to the baseline condition. Importantly, this improvement of relief frequency showed little or no continuation in relief behaviour into week 3 (follow-up), as relief duration decreased significantly again when the feedback system was removed. Non-adherence with PU prevention strategies, such as relief behaviour, is a known issue in the SCI population [32–35]. For some of these individuals, the non-compliance with prevention strategies is possibly related to a lack of incentives to maintain healthy behaviour and a misconception about PU risk [36]. For example, more than 80% of a sample of people with SCI who had experienced a previous PU did not believe they were at risk of future PUs [37]. Many of the recommendations for PU prevention, such as performing pressure reliefs, require understanding, cooperation, and initiative, and evidence from the behavioral medicine literature indicates that especially those recommendations involving lifestyle changes are associated with poor adherence [38–40].

Therefore, one could claim that feedback on sitting behaviour for wheelchair users with SCI should be provided over prolonged periods of time in order to maintain high-quality relief activities. Hence, a constant use of the feedback system is expected to improve sitting behaviour routine and ultimately be advantageous for PU prevention.

Seminal research into PU aetiology using animal models has highlighted that the damaging effects of pressure are related to both its loading magnitude and duration [41, 42]. Moreover, these studies indicated that magnitude and duration were inversely related, meaning that tissues can withstand higher loading for relatively short periods of time compared to lower tissue loads for longer periods. Based on these findings, we not only aimed at improving relief frequency but also its duration. In this regard, the results of our study demonstrate that all participants with short relief durations in the first measurement week, i.e., <100% of the OT’s recommendations, increased their relief duration by responding to the feedback. Similar to relief frequency, this effect could not be transferred into week 3 (follow-up, no feedback).

Overall, the feedback system was well received by all participants. They all reported increased motivation to perform pressure relief activities on a regular basis. Conclusively, a transient improvement in relief behaviour, i.e., increased relief frequency and duration, could be demonstrated by employing the new feedback system. In how far this translates into a clinically relevant reduction in the risk for PU development remains, however, unclear at this point. On one hand, several studies show no relationship between pressure relief behaviour and PU prevalence in the SCI population [12, 43, 44]. However, each of these studies relied on self-report measures of pressure relief practices, even though self-reported behaviour is known to be highly unreliable [31]. On the other hand, positive effects of a variety of pressure relief activities on actual pressure distribution, blood flow, and tissue oxygenation have been reported in healthy controls [45] as well as individuals with SCI [46, 47]. In addition, Sonenblum and Sprigle [30] reported that wheelchair users with no PU history perform significantly more weight shifts than individuals with a history of recurrent pressure injuries. However, their study failed to find a connection between PU history and the relief frequency, which was possibly due to the fact that pressure reliefs were performed less than once every 3 h in both groups.

Therefore, a longitudinal follow-up study to investigate the long-term effects of improved pressure relief behaviour on PU development in a large cohort of wheelchair users is clearly warranted. While the impact of PU history on the intrinsic motivation for using a suitable feedback, application remains to be investigated, our study provides clear evidence that feedback on relief form, frequency and duration, provided to the user in a suitable manner, is able to improve routine relief behaviour.

## Limitations

We would like to emphasize that this is a pilot study including only nine participants with rather heterogeneous SCI. Therefore, the generalizability of our findings to a wider population needs to be addressed with caution. The potential bias due to the reported conflict of interest has been minimized by strictly following scientific standards as well as giving the primary lead of this study to the Balgrist team (MH, UA, AC), sharing no conflict of interest.

## Data archiving

The datasets generated and/or analysed during the current study are available from the corresponding author on reasonable request.
